# The Heating and Lighting of Hospital Wards

**Published:** 1909-02-06

**Authors:** 


### HOSPITAL ADMINISTRATION.

### CONSTRUCTION AND ECONOMICS.

### THE HEATING AND LIGHTING OF HOSPITAL WARDS.

#### III.—THE PLENUM SYSTEM.

The method described in the last article of heating the air from outside by allowing it to pass over the surfaces of radiators, has led naturally to the question, Cannot the whole of the air in a hospital be heated? In the Plenum system the problems of heating and ventilation are solved together. The air to be breathed in the wards and in all parts of the hospital governed by the system is cleansed from dirt such as exists in our smoky towns, cooled in hot weather, warmed in cold weather, forced through the wards, corridors, etc., to be heated and ventilated, and carried off into the outer atmosphere again, at a point as far removed as possible from that at which it entered. The usual arrangement includes a shaft taken down from the outer atmosphere to the basement, and connected with a hole, usually circular, to accommodate a fan, cut in the outer wall of the building. At the bottom of the shaft, outside the building, are placed cleaning and cooling apparatus. In hospitals which have adopted the Plenum system there is usually a mat of some fibrous material, kept continually wetted with clean fresh water, through which the air to be used in the hospital is obliged to pass. There are also grids of steam pipes provided outside the mat to keep the water employed in washing the air, in the liquid condition in frosty weather. In passing through the mat the air delivers up any dirt it may carry, and in our large towns this is a considerable quantity. It is also cooled to a temperature a few degrees above that of the water employed, and in the process of cooling it is made to deposit any surplus moisture it carries, an important point in muggy weather. The circular aperture in the wall of the building is filled with a fan, and the air from the shaft is sucked by this through the mat described, and delivered to ducts leading to the different wards or other rooms to be warmed and ventilated. At the entrance of each duct there is a grid of steam pipes, arranged so that any temperature may be obtained that is desired, higher temperature being obtained by allowing steam at a higher pressure to pass into the grid and *vice versa*. The ducts are large near the air entrance; smaller ducts branch off to feed different groups of wards and individual wards. The air is usually led into the ward by openings controlled by sliding valves at about 8 feet above the floor. In some cases, as in the Birmingham General Hospital, the air enters by ducts placed in front of the windows, and is given an upward motion. The air passes out of the wards or the rooms to be warmed and ventilated, sometimes by the ordinary chimneys, but more frequently by ducts whose openings are on the same side as those by which the air entered and a few inches above the floor. Ducts lead from these openings to a shaft which carries the air off to the outer atmosphere.

Another method of cooling and cleaning the air is employed in the Manchester School of Technology. The air is led down a shaft, as described above, and it is exposed for a large portion of the depth of the shaft to a very fine spray of water. A fine mist is created for a large portion of the shaft; the entering air has to pass through the mist and is cooled; any dirt that is present is deposited at the bottom of the shaft. When the present writer visited the school, by the courtesy of the Principal, the engineer brought a spade-full of dirt from the bottom of the shaft, deposited that day.

One great advantage of the Plenum system is the complete control of the warming and ventilation and the complete absence of dust, and of any requirement for attendance upon any apparatus, such as radiators, etc., in the wards. On the other hand, in order that the system may be carried out successfully, it is absolutely necessary that all ordinary means of ventilation, such as open windows, should be suppressed. An open window in any ward may interfere with the proper circulation of the air current. Another disadvantage, which is probably only apparent, is that wherever the Plenum system has been adopted in hospitals complaint has been made that, while it is apparently good for certain wards, it is not good for others, and that both nurses and resident staff strongly object to it in their quarters. The common complaint from medical men and nurses is that where the Plenum system is used in their quarters they have a feeling of staleness, which is not present under ordinary natural conditions. There appears to be a feeling that something has been taken out of the air, and that expression has been used to the writer by eminent men who have considered the question. The writer suggests that the explanation of the whole of the above objections, the staleness, etc., lies in the fact that the temperature of the air in the whole of the wards and that of the quarters of the resident staff is maintained at a figure higher than would be obtained at certain times of the year under ordinary conditions. The remedy evidently is to regulate the temperature according to the requirements of the particular rooms to be warmed and ventilated. At the Manchester School of Technology a system is employed in which a supply of cold air and one of warmed air enter the room together, and there is a valve, under the control of those in the room, by which the quantity of warm or cold air can be altered. The engineer finds that the requirements of the different class-rooms vary very much. One professor's throat may require the air of a certain temperature and a certain humidity, while the professor in the next class-room requires both a little different. With the above means the matter is controlled by the professors themselves. It can also be regulated automatically by thermostats.

